# Rheopheresis, but Not Phlebotomy, Improves Cerebral Vascular Response to Hypercapnia and Neuronal Activation

**DOI:** 10.3390/biomedicines14030718

**Published:** 2026-03-20

**Authors:** Dóra Sulina, Szonja Krisztina Rab-Bábel, Ádám Varga, Ádám Attila Mátrai, Kristóf Gál, Pál Soltész, Norbert Németh, László Oláh

**Affiliations:** 1 Department of Neurology, Faculty of Medicine, University of Debrecen, 4032 Debrecen, Hungary; babel.szonja@med.unideb.hu (S.K.R.-B.); olah@med.unideb.hu (L.O.); 2 Department of Operative Techniques and Surgical Research, Faculty of Medicine, University of Debrecen, 4032 Debrecen, Hungary; varga.adam@med.unideb.hu (Á.V.); matrai.adam@med.unideb.hu (Á.A.M.); nemeth@med.unideb.hu (N.N.); 3 Division of Angiology, Department of Internal Medicine, Faculty of Medicine, University of Debrecen, 4028 Debrecen, Hungary; gal.kristof@med.unideb.hu (K.G.); soltesz.pal@med.unideb.hu (P.S.)

**Keywords:** rheopheresis, phlebotomy, cerebral circulation, transcranial Doppler, hemorheology, neurovascular coupling, cerebral vasoreactivity

## Abstract

**Background/Objectives**: Several therapeutic approaches, including phlebotomy and rheopheresis, are used to improve hemorheological parameters. While the effects of phlebotomy on cerebral circulation have been described, the impact of rheopheresis on cerebral hemodynamics remains poorly understood. This study primarily aimed to evaluate the within-group effects of phlebotomy and rheopheresis on cerebral blood flow velocity changes evoked by neuronal activation and on cerebrovascular reactivity in patients with elevated hematocrit or hyperviscosity, respectively. **Methods**: In our present study, we used transcranial Doppler to examine the effects of phlebotomy (*n* = 11) and rheopheresis (*n* = 9) on cerebral hemodynamics in patients with elevated hematocrit and hyperviscosity, respectively. Measurements included resting flow velocity (FV) in the posterior cerebral artery (PCA) and middle cerebral artery (MCA), the visually evoked FV response in the PCA (neurovascular coupling) and the hypercapnia-induced FV response in the MCA (cerebral vasoreactivity). In addition to flow velocity data, visual evoked potential (VEP) parameters were also recorded to assess neuronal activation. **Results**: Phlebotomy significantly reduced hematocrit and hemoglobin levels, while rheopheresis led to a significant decrease in both blood and plasma viscosity. Although we observed no differences in resting FV values before and after either intervention, the FV increase in response to visual stimulation and hypercapnia was greater after rheopheresis than before, whereas no such difference was observed following phlebotomy. VEP parameters remained similar before and after both phlebotomy and rheopheresis. **Conclusions**: Our data indicate that rheopheresis reduces blood and plasma viscosity in patients with hyperviscosity and leads to a significant improvement in cerebral vasoreactivity and neurovascular coupling, without affecting VEP parameters. The improvement in cerebral vasoreactivity, but no changes in VEP parameters, suggests that the improved FV response to visual stimulation after rheopheresis is most likely caused by better vascular response rather than improved neuronal activation.

## 1. Introduction

Blood viscosity is determined by both cellular elements (e.g., hematocrit, red blood cell deformability, and aggregation) and plasma components. Plasma viscosity primarily depends on the concentration of plasma proteins such as fibrinogen, immunoglobulins, acute-phase proteins, inflammatory cytokines, and lipoproteins [1]. Elevations in either the cellular (e.g., polycythemia, leukemia, thrombocytosis) or acellular (e.g., hypergammaglobulinemia, hypertriglyceridemia) components can lead to hyperviscosity [2,3]. This condition adversely affects microcirculation, reduces tissue perfusion, and increases the risk of thrombotic events, cardiovascular and cerebrovascular diseases [4,5,6]. Hyperviscosity has also been linked to cognitive impairment [7] and abnormalities in brainstem auditory evoked potentials [8], which were assumed to be caused by cerebral microcirculatory disturbances.

Therapeutic approaches to improve hemorheology include hemodilution with or without phlebotomy, plasmapheresis, and rheopheresis—a selective double-filtration plasmapheresis. Rheopheresis targets high-molecular-weight plasma components (fibrinogen, von Willebrand factor, α2 macroglobulin, IgM/IgG type antibody), lipoproteins (lipoprotein (a), low-density lipoprotein), various pathological antibodies (antineutrophil cytoplasmic antibody, antiphospholipid antibody) and inflammatory cytokines, which increase viscosity, promote atherosclerosis and thrombosis, and induce inflammatory processes. While therapeutic phlebotomy is approved for hemochromatosis, polycythemia vera, erithrocytosis and porphyria cutanea tarda, rheopheresis has been increasingly applied in hyperviscosity syndrome, severe hypertriglyceridemia, peripheral arterial disease, age-related macular degeneration, sensorineural hearing loss, inflammatory vascular diseases, and diabetes-induced lower-extremity ulcers [9,10].

Historically, hemorheological abnormalities were implicated in the pathogenesis of cerebral ischemia [11,12,13,14,15]. Hemodilution with or without phlebotomy was shown to enhance cerebral blood flow (CBF) in ischemic stroke patients [16,17,18] and reduce infarct volume in experimental stroke models [19,20]. In polycythemia patients, phlebotomy reduced hematocrit and whole-blood viscosity, improving cerebral perfusion and reducing the rate of cardiovascular events and death [21,22], a Cochrane review of randomized controlled trials (*n* = 4174) failed to confirm a clinical benefit of hemodilution in acute ischemic stroke [23]. In contrast to hemodilution, only one study has examined the clinical effects of rheopheresis in ischemic stroke, involving 33 stroke patients without confirmed hyperviscosity [24,25]. Importantly, prior trials of phlebotomy or rheopheresis have not stratified patients based on elevated hematocrit or blood viscosity. Therefore, the impact of these interventions on cerebral hemodynamics in patients with increased hematocrit or confirmed hyperviscosity remains unknown.

Although rheopheresis is generally accepted to improve microcirculation [26], no data are available on its effects on cerebral circulation. Given the association between hyperviscosity and cognitive dysfunction [7], and its presumed contribution to cerebral ischemia, investigating cerebral hemodynamic changes following rheopheresis in patients with hyperviscosity may reveal new therapeutic options in this special patient group.

In this study, we aimed to assess whether phlebotomy or rheopheresis improves cerebral blood flow velocity (FV) and cerebral blood flow velocity response to neuronal activation (neurovascular coupling) in patients with erythrocytosis (*n* = 11) or hyperviscosity (*n* = 9). Using transcranial Doppler, we measured resting FV in the posterior cerebral artery (PCA) and middle cerebral artery (MCA), and visually evoked flow velocity responses in the PCA before and after intervention. We also evaluated cerebral vasomotor reactivity to hypercapnia and visual evoked potentials (VEPs) to distinguish between vascular and neuronal contributions to any observed changes in flow velocity response induced by neuronal activation.

## 2. Materials and Methods

This prospective study was conducted between 2018 and 2022 at the Department of Neurology, University of Debrecen.

### 2.1. Study Participants

Eligible participants were adults aged 18–80 years who provided written informed consent. Patients with impaired best corrected visual acuity of less than 1.0 in either eye were excluded. Additional exclusion criteria included malignancy, untreated hypertension, or untreated diabetes mellitus. Patients with untreated hypertension or untreated diabetes mellitus were excluded because both conditions are known to impair cerebral vasoreactivity, endothelial function, and neurovascular coupling independently of hemorheological status [27,28]. Including untreated cases could have introduced significant confounding effects on TCD-derived hemodynamic parameters. Participants were assigned to either the phlebotomy group or the rheopheresis group based on clinical indication and laboratory findings.

The phlebotomy group (*n* = 11) included five patients diagnosed with polycythemia vera, all with a confirmed *JAK2* mutation, and six patients with erythrocytosis, defined as hemoglobin > 165 g/L in men or >160 g/L in women, or hematocrit > 49% in men or >48% in women. Of the six patients with erythrocytosis, one had a *VHL* RS779805 gene mutation, while the remaining five had no identified genetic cause.

The rheopheresis group consisted of 9 patients with hyperviscosity-related disorders requiring rheopheresis. Eight patients had diabetes-induced lower extremity ulcers associated with hyperviscosity, and one had severe hypertriglyceridemia (triglyceride level > 11.3 mmol/L).

### 2.2. Study Protocol

Medical history was recorded, and all participants underwent a detailed neurological examination, standard laboratory testing, and a series of neurovascular and neurophysiological assessments using transcranial Doppler (TCD) and visual evoked potential (VEP) examinations. The neurovascular evaluation involved measurement of resting flow velocity (FV) in both the posterior cerebral artery (PCA) and the middle cerebral artery (MCA). Neurovascular coupling was assessed by monitoring the visually evoked FV changes in the PCA, while cerebral vasomotor reactivity was evaluated by calculating the breath-holding index (BHI) in the MCA. Visual evoked potentials (VEPs) were also recorded, and P100 wave parameters were analyzed to assess visual cortex activation. All TCD and VEP examinations were performed by the same experienced examiner to ensure consistency.

The examinations were performed in a quiet, dark room at about 22 °C while the patients were sitting comfortably.

In the phlebotomy group, which included patients with increased hemoglobin and/or hematocrit values, assessments were performed 1 day prior to phlebotomy and repeated 2 days following the intervention.

In the hyperviscosity group, 2 patients underwent one session, while 7 patients had two sessions of rheopheresis between the control (pre-treatment) and follow-up (post-treatment) examinations. The assessment protocol in this group was identical to that of the phlebotomy group: transcranial Doppler and neurophysiological evaluations were conducted one day before and two days after the rheopheresis treatment. To minimize diurnal variation, all measurements—both pre- and post-treatment—were performed between 7:00 and 9:00 a.m. The current study protocol was approved by the Ethics Committee of the Hungarian Medical Research Council (Registration number: 26426–8/2018/EÜIG), and written informed consent was obtained from all participants prior to inclusion in the study.

### 2.3. Functional TCD Study

To evaluate neuronal activation-induced flow responses, referred to as neurovascular coupling, bilateral insonation of the P2 segment of the PCAs at a depth of 58–60 mm was performed using two 2 MHz probes. The probes were fixed over the temporal acoustic windows using an individually fitted headband. Peak systolic (PSV) and time averaged mean (TAMV) blood flow velocities were measured using a Multidop T Doppler system (DWL, Singen, Germany). Vessel identification followed the method described by Fujioka and Donville [29]. Given that vasodilatory stimuli exert a greater effect on mean FV values, while peak systolic FV values are less susceptible to Doppler-related artifacts [30], both TAMV and PSV were recorded and analyzed.

**Figure 1:**
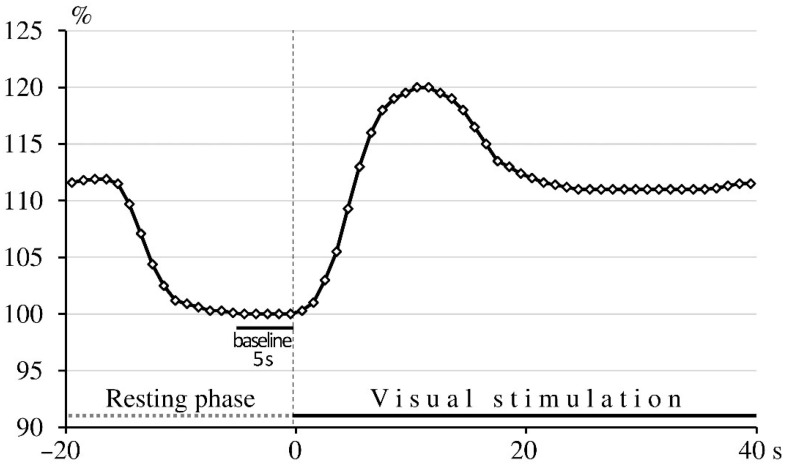
Schematic representation of the visually evoked flow response in the posterior cerebral artery. Following a brief delay after the onset of visual stimulation (time 0 s), the relative cerebral blood flow velocity rises rapidly, shows a slight overshoot, and then stabilizes at a constant but higher level than the baseline.

A high-contrast, black-and-white checkerboard pattern-reversal stimulation was used as a stimulation paradigm, with a 32 × 32 checkerboard pattern and 2 Hz reversal frequency, generated on a computer screen. The stimulation protocol consisted of 10 cycles, each comprising a 20 s resting phase followed by a 40 s stimulation phase. During the resting phase, participants were instructed to close their eyes; during the stimulation phase, they focused on a central red fixation dot on the checkerboard. The FV data of 10 cycles within one patient were averaged. For averaging procedures beat-to-beat intervals of cerebral blood FV data were interpolated linearly with a “virtual” time resolution of 10 ms. To ensure independence from the insonation angle and to enable comparisons between patients, absolute data were converted into relative changes in cerebral blood FV in relation to baseline. Baseline was defined as the average FV during the final 5 s of the resting phase, immediately preceding stimulation. At the beginning of the visual stimulation, cerebral blood FV increased rapidly after a brief latency, reaching a peak (the “overshooting” phase), followed by a slight decline and stabilization at a constant but higher level than the baseline (the “plateau” phase; Figure 1). The maximum relative increase in FV during the stimulation phase was identified and used for further analysis. Relative FV was expressed as a percentage of baseline.

### 2.4. Breath-Holding Index

The breath-holding test was used to assess the cerebral vasoreactivity [31,32]. After completing the measurements of FV changes in the PCAs, the position of the TCD probes was adjusted bilaterally to insonate the M1 segment of the MCA at a depth of 50 mm. Participants were instructed to take a normal (non-forced) inspiration and then hold their breath for 30 s, avoiding any Valsalva maneuver. Systolic and mean blood flow velocities were recorded before (baseline) and during the breath-holding period. The breath-holding index (BHI) was calculated by dividing the percentage increase in mean FV relative to baseline by the duration of the breath-holding period (30 s) [33]. BHI values obtained from the left and right MCAs were averaged for each subject and used for statistical analysis.

### 2.5. Visual Evoked Potential

For visual evoked potential recordings over the visual cortex, standard 10 mm silver/silver chloride cup electroencephalography electrodes were used and fixed by contact paste on the scalp. Three active electrodes were placed according to the International 10/20 system as recommended by the International Society for Clinical Electrophysiology of Vision (ISCEV): the Oz electrode was placed at 10% of the inion-nasion distance above the inion, and the O1 and O2 electrodes were placed 10% of the head circumference laterally to the left and right, respectively. The Fz reference electrode was placed 20% of the inion-nasion distance anterior to the Cz (vertex). The ground was placed at the vertex (Cz). The filter setting was 1–100 Hz. The analysis time was 600 ms, and the sensitivity was 10 mV per division. VEPs at three montages (Oz–Fz, O1–Fz and O2–Fz) were recorded. Two hundred responses evoked by high-contrast, black-and-white checkerboard pattern reversal stimulation were averaged (Neuron-Spectrum-4/EPM, Neurosoft, Ivanovo, Russia), and latencies and amplitudes of the P100 waves were calculated.

### 2.6. Laboratory Examinations

Blood samples were collected prior to transcranial Doppler (TCD) and neurophysiological assessments on both the control (pre-treatment) and follow-up (post-treatment) days, i.e., one day before and 2 days after phlebotomy or rheopheresis. In addition to hemorheological measurements, a comprehensive panel of hematological parameters was also evaluated.

#### Hemorheological Parameters

The whole-blood and plasma viscosity were measured with the Hevimet-40 capillary viscometer (Hemorex Kft., Budapest, Hungary). The hematological parameters were measured with the Sysmex K-4500 microcell counter (Sysmex Corporation, Kobe, Japan).

Red blood cell deformability, which describes the ability of the red blood cells to change their shapes, was measured with using the LoRRca MaxSis Osmoscan ektacytometer (RR Mechatronics, Hoorn, The Netherlands). Two measurement methods were applied. The first one assessed red blood cell deformability under increasing shear stress (analysed parameters: elongation index at a shear stress of 3 Pascal [EI 3Pa], maximal elongation index [EImax], and shear stress at the half of the maximal elongation index [SS1/2]). The second protocol evaluated deformability under varying osmolality (50–500 mOsm/kg) resulting in an elongation index–osmolality curve. Measuring the elongation index at different osmolalities provides different deformability parameters, including minimal elongation index, i.e., the lowest deformability value at low osmolality [EImin], the maximal elongation index reflecting the highest deformability [EImax], and the elongation index in the hyper-osmotic environment [EI hyper]. Corresponding osmolality values were also determined: the osmolality at which hemolysis begins or deformability is minimal [Omin], the osmolality where maximal deformability occurs [O(EI)max], and the osmolality at which elongation index in the hyperosmotic region is measured [Ohyper]. The area under the elongation index–osmolality curve [area] was calculated as an overall index of red blood cell deformability across the entire osmotic range [34,35].

Red blood cell aggregation, which is a reversible connection between the erythrocytes, was also measured using two methods. The first method was based on the principle of light transmission (four index parameters were detected: M5s, M1 5s, M10s, and M1 10s) and was measured with the Myrenne MA-1 aggregometer (Myrenne GmbH, Roetgen, Germany). According to the operating principle of the device, the red blood cells in the sample are first disaggregated (at a shear rate gradient of 600 s^−1^), after which the shear rate gradient is suddenly reduced to zero (stasis, M-mode) or to a low shear stress value (3 s^−1^, M1-mode), allowing the red blood cells to re-aggregate. The device then measures the intensity of light passing through the sample at the 5th and 10th seconds of the aggregation process. For the other method the LoRRca MaxSis Osmoscan ektacytometer was used, which measurement was based on light-reflectance during the aggregation process. The aggregation index [AI] represents the percentage ratio of light transmission at 10 s compared to the full range of change during aggregation. The amplitude [amp] indicates the total change in transmitted light intensity throughout the aggregation process, i.e., the difference between baseline and plateau values. The half-time of aggregation [t1/2] shows the time required to reach 50% of the maximal amplitude of light transmission, while the slope-index at half amplitude [isc1/2] reflects the rate of change of the aggregation curve at the time point corresponding to half of the maximal amplitude [34,36].

### 2.7. Rheopheresis

The rheopheresis treatments were performed in the Intensive Care Unit and the Therapeutic Apheresis Center of the Department of Internal Medicine, Clinical Center, University of Debrecen. Prior to each session, patients received a 500 mL infusion of physiological saline.

After canulation of two peripheral veins, blood plasma was separated from blood cells using the Art Universal system. Separated plasma was pumped into a column containing a MONET filter (Membrane filtration Optimized Novel Extracorporal Treatment) (Fresenius SE and Co., KGaA, Bad Homburg vor der Höhe, Germany) for elimination of plasma proteins and macromolecules larger then 250–300 kDa. The filtered plasma was subsequently recombined with the cellular components and returned to the patient’s circulation via peripheral venous cannula. The volume of the plasma to be filtrated was calculated according to the patient’s body weight, using a standard of 40 mL per kilogram. Anticoagulation during the procedure was maintained with citrate.

### 2.8. Phlebotomy

The phlebotomy treatments were performed in the Hematology Center of the Department of Internal Medicine, Clinical Center, University of Debrecen. Patients were positioned supine during the procedure. Phlebotomy involved the removal of 300 mL of blood through a peripheral vein.

### 2.9. Statistical Analysis

The absolute and relative FV data, cerebral vasoreactivity values (BHI), and VEP latencies and amplitudes, measured on the right and left sides were averaged, and the averaged data were used for analysis.

Normality of continuous variables was assessed using the Shapiro–Wilk test.

Laboratory data, including hemorheological and blood count parameters, absolute baseline flow velocities in the MCA and PCA, maximum increase in relative flow velocities (relative TAMV and PSV) evoked by visual stimulation, breath-holding index and VEP P100 amplitudes and latencies before and after rheopheresis or phlebotomy, were compared by paired *t*-test.

Analysis of variance (ANOVA) was used to compare age, systolic and diastolic blood pressure, pulse rate, and the maximal increases in relative flow velocities (relative TAMV and PSV) evoked by visual stimulation between the phlebotomy and rheopheresis groups. Categorical variables, including prevalence of stroke risk factors, were compared between groups using the chi-square test.

To evaluate treatment effects on visually evoked cerebral blood flow responses, repeated measures analysis of variance (ANOVA) was applied. This analysis compared relative FV changes (i.e., relative FV time courses) during the stimulation phase before and after treatment within each group (phlebotomy or rheopheresis) and between groups. The results of the repeated measures analysis of variance were shown by the group main effect and the group with time-of-measurement interaction. The group main effect showed whether there was a significant difference in flow velocities averaged over the 40 s active period during visual stimulation before and after treatment within each group (rheopheresis or phlebotomy), and between the two groups. The group with time-of-measurement interaction indicated whether the pattern of FV changes over time was different between the two curves. A non-significant interaction indicated that the FV or pulsatility index time courses before and after treatment were parallel.

Data were expressed as means ± standard deviation. A difference of *p* ≤ 0.05 was considered statistically significant.

## 3. Results

We conducted a self-controlled follow-up study involving 11 patients in the phlebotomy group and 9 patients in the rheopheresis group. Demographic characteristics, prevalence of stroke risk factors and physiological data are shown in Table 1. Patients in the rheopheresis group were significantly older than those in the phlebotomy group (paired *t*-test, *p* < 0.05). None of the participants had a history of stroke. The majority of patients in the rheopheresis group (8 out of 9) and all patients in the phlebotomy group were male. Diabetes mellitus and hypercholesterolemia were significantly more common in the rheopheresis group compared to the phlebotomy group (chi-square test, *p* < 0.05 for both conditions). There were no significant differences in blood pressure or pulse rate between the two groups, nor before and after the intervention within either group (Table 1).

### 3.1. Effects of Phlebotomy and Rheopheresis on Hemorheological Parameters

The effects of rheopheresis and phlebotomy on laboratory parameters are presented in Table 2. Phlebotomy, involving the removal of 300 mL of blood, led to a significant decrease in red blood cell count, hemoglobin concentration, and hematocrit levels. In contrast, rheopheresis significantly reduced whole-blood viscosity, plasma viscosity, and whole-blood viscosity corrected to a normal hematocrit of 40%.

The hematocrit-to-viscosity ratio, an indicator of red blood cell oxygen transport efficiency, increased significantly following rheopheresis, but not after phlebotomy. Furthermore, while phlebotomy had no measurable effect on red blood cell deformability or aggregation, rheopheresis significantly reduced certain parameters of red blood cell aggregability, as indicated by a decrease in aggregation of erythrocytes at a zero shear rate value in the 5th second (M5), as well as decreases in aggregation index (AI), and aggregation amplitude (Amp) values compared to the pretreatment values.

### 3.2. Transcranial Doppler Studies

In two patients from the phlebotomy and one patient from the rheopheresis group, only unilateral recordings of PCA flow parameters could be obtained due to technical reasons. For these participants, unilateral PCA FV data were used in the statistical analysis.

#### 3.2.1. Baseline Flow Velocity Values in the PCA and MCA Before and After Phlebotomy or Rheopheresis

Baseline absolute peak systolic flow velocity (PSV) and time-averaged mean flow velocity (TAMV) recorded in the PCA, as well as TAMV measured in the MCA, did not differ significantly before and after intervention in either the phlebotomy or rheopheresis group (Table 3).

#### 3.2.2. Effects of Phlebotomy and Rheopheresis on Neurovascular Coupling: Visually Evoked Flow Velocity Parameters in the PCA

**Figure 2:**
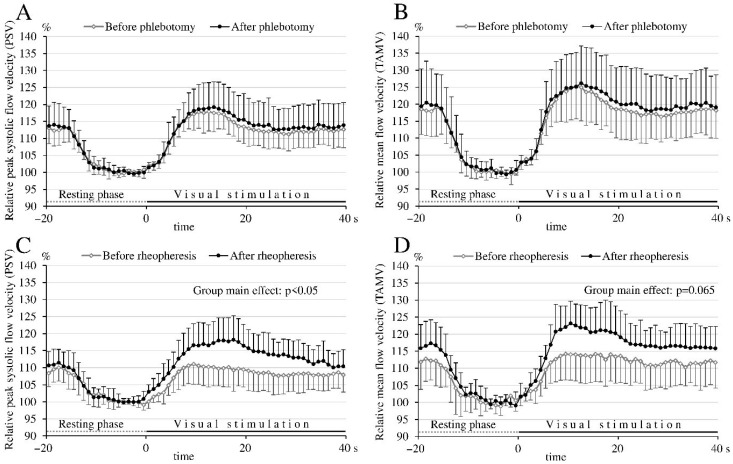
Relative time courses of peak systolic (PSV) and time-averaged mean flow velocities (TAMV) before and after phlebotomy (panels (**A**) and (**B**), respectively) and before and after rheopheresis (panels (**C**) and (**D**), respectively). To improve clarity and avoid overlap of the standard deviation (SD) bars, SDs are shown upward for the “after phlebotomy” and “after rheopheresis” curves, and downward for the “before phlebotomy” and “before rheopheresis” curves. The increase in flow velocity during visual stimulation was greater after rheopheresis than before, as indicated by a significant main effect (*p* = 0.03) in peak systolic flow velocities and a trend (*p* = 0.07) in mean flow velocities (repeated-measures ANOVA). In contrast, no such difference was observed in peak-systolic and mean flow velocities (*p* = 0.64 and *p* = 0.68, respectively) before and after phlebotomy.

To compare hemodynamic changes between patients, absolute FV data before and after intervention were normalized by calculating relative FV changes relative to the appropriate baseline values. Analysis of the effect of neuronal activation on these relative FV changes revealed a significant increase in FV during visual stimulation compared to baseline, both before and after phlebotomy or rheopheresis (Figure 2).

Analyzing the visually evoked relative FV time courses between the control (pre-treatment) and follow-up (post-treatment) periods in the phlebotomy group, repeated measures ANOVA revealed no significant group main effect (*p* > 0.05 for both the PSV and TAMV values), indicating that the relative FV values during visual stimulation were comparable before and after phlebotomy (Figure 2A,B). The group with time of measurement interaction was also not significant (*p* > 0.05), suggesting that the pattern of FV changes over time was similar before and after phlebotomy.

In contrast, repeated measures ANOVA revealed a significant group main effect (*p* < 0.05) and a significant group with time of measurement interaction (*p* < 0.01) in the visually evoked relative PSV time courses before and after rheopheresis (Figure 2C). For the TAMV time courses, repeated measures ANOVA showed a trend toward higher relative FV values after rheopheresis (group main effect: *p* = 0.065) along with a significant group with time of measurement interaction (*p* < 0.01) (Figure 2D). The higher relative flow velocities after rheopheresis indicate that the flow velocities evoked by visual stimulation increased more after rheopheresis compared to the control (pre-treatment) period. The significant group with time of measurement interaction suggests that the pattern of FV changes differed before and after rheopheresis.

Analysis of the maximum changes in relative FV values before and after phlebotomy showed that the maximum increases in both PSV and TAMV during visual stimulation were similar before and after the treatment (*p* > 0.05). In contrast, following rheopheresis, the maximum increases in relative PSV and TAMV were significantly greater than in the control period prior to treatment (Table 4).

Although our study was not initially designed to compare the visually evoked FV data between the two groups, the marked differences in the effects of rheopheresis and phlebotomy prompted us to explore intergroup comparisons before and after treatment. Repeated measures ANOVA revealed a significant group main effect in the visually evoked relative TAMV and PSV time courses between the two groups (i.e., rheopheresis and phlebotomy) before treatment (*p* = 0.04 and *p* = 0.03, respectively), indicating that visual stimulation induced smaller increases in relative FV in the rheopheresis group compared to the phlebotomy group. After treatment, however, no significant differences were observed in neuronal activation-induced FV responses between the groups (*p* = 0.46 for TAMV and *p* = 0.85 for PSV), reflecting an improvement in visually evoked FV increases within the rheopheresis group after the intervention.

The maximum relative FV response to visual activation was significantly lower in the rheopheresis group before treatment than in the phlebotomy group before venesection (*p* = 0.03 for TAMV and *p* = 0.02 for PSV). However, after treatment, the enhanced visually evoked flow velocity response in the rheopheresis group eliminated this difference between the two groups (*p* = 0.44 for TAMV and *p* = 0.67 for PSV).

#### 3.2.3. Effect of Phlebotomy and Rheopheresis on Neuronal Activity: VEP Parameters

There were no significant changes in the latency or amplitude of the VEP P100 wave following phlebotomy. Specifically, latency was 118.0 ± 6.4 ms before and 116.1 ± 6.0 ms after phlebotomy (*p* = 0.09), while amplitude was 5.2 ± 2.5 μV before and 6.1 ± 3.3 μV after the procedure (*p* = 0.09). Similarly, VEP parameters were also comparable before and after rheopheresis: P100 latency was 120.2 ± 5.1 ms before and 120.4 ± 6.6 ms after rheopheresis (*p* = 0.23), and amplitude was 4.2 ± 1.1 μV before and 4.3 ± 1.3 μV after the intervention (*p* = 0.80).

#### 3.2.4. Effect of Phlebotomy or Rheopheresis on Cerebral Vasoreactivity: Breath-Holding Index Parameters

The increase in blood FV in the MCA induced by 30 s of breath-holding was similar before and after phlebotomy (0.94 ± 0.21%/s before vs. 1.06 ± 0.22%/s after; *p* = 0.10). In contrast, the FV increment was significantly higher after rheopheresis (1.24 ± 0.42%/s) than before the treatment (0.96 ± 0.37%/s; *p* = 0.04).

## 4. Discussion

In the present study, we primarily aimed to investigate the within-group effects of phlebotomy and rheopheresis on cerebral hemodynamics, including resting FV in the PCA and MCA, the visual stimulation-evoked FV response in the PCA, and the hypercapnia-induced cerebral vasomotor reactivity in the MCA. While phlebotomy led to reductions in red blood cell count, hematocrit, and hemoglobin levels, rheopheresis decreased the whole blood and plasma viscosity. Neither phlebotomy nor rheopheresis resulted in significant changes in the resting flow velocities measured in the MCA or PCA. However, rheopheresis augmented both the visual stimulation evoked FV response in the PCA, and the hypercapnia induced a flow velocity increase in the MCA, indicating improved neurovascular coupling and cerebral vasomotor reactivity, respectively, with no significant changes observed following phlebotomy. As visual evoked potential (VEP) amplitudes, used to assess neuronal activation, remained unchanged before and after rheopheresis, the increased FV responses to visual stimulation after rheopheresis are attributed to improved vascular reactivity rather than enhanced neuronal activation. To the best of our knowledge, this is the first human study to demonstrate that rheopheresis improves both neurovascular coupling and cerebral vasoreactivity. Further research is needed to determine whether these effects of rheopheresis are solely due to improved blood and plasma viscosity or also involve other molecular mechanisms.

### 4.1. Effects of Phlebotomy and Rheopheresis on Blood Count and Rheological Parameters

The removal of 300 mL of blood resulted in a modest reduction in red blood cell count, hemoglobin concentration, and hematocrit value, measured two days after phlebotomy. However, this intervention did not alter whole-blood viscosity, plasma viscosity, red blood cell deformability, or aggregation.

Our findings are similar to those of Murugesan et al. [37], who reported a 7.8 g/L decrease in hemoglobin and a 2.4% reduction in hematocrit immediately after the removal of approximately 304 mL of blood. A more pronounced decrease in hematocrit (4%) was observed by Kong et al. [38] following the removal of an average of 458 mL of blood. The smaller hematocrit reduction observed in our study (1.8 ± 0.9%) may be attributed to the modest volume of blood removed and the two-day interval between phlebotomy and blood sampling, which allowed partial hematological compensation.

Rheopheresis did not significantly change hemoglobin or hematocrit levels; however, unlike phlebotomy, it markedly reduced both whole-blood and plasma viscosity and improved certain parameters of red blood cell aggregation.

### 4.2. Baseline Flow Velocity Data Before and After Phlebotomy or Rheopheresis

Baseline FV parameters were recorded in the MCA prior to the breath-holding test and in the PCA at the end of the resting phase, just before the onset of the visual stimulation phase (Figure 1). Based on the observed reductions in hematocrit following phlebotomy and in blood viscosity after rheopheresis, we expected an increase in FV after the interventions. However, no significant changes in absolute FV parameters were detected in either the MCA or PCA following either treatment. It should be noted as a limitation that the TCD probes were removed after the first measurements before interventions and were mounted again two days later for the post-treatment assessments following phlebotomy or rheopheresis. Efforts were made to maintain consistency by using the same acoustic bone window and insonation depth for pre- and post-treatment recordings of absolute FV data. Nevertheless, potential variability in measurement conditions, including differences in the insonation angle or changes in physiological parameters, may have affected the comparability of the control and follow-up data. In addition to potential measurement variability, the lack of significant changes in resting flow velocity despite alterations in hemorheological parameters may reflect compensatory regulatory processes maintaining stable cerebral blood flow. While classical cerebral autoregulation primarily describes the response of cerebral resistance vessels to changes in perfusion pressure, microvascular tone may also adapt to changes in blood viscosity, thereby contributing to the stability of resting flow velocities observed in our study [39,40].

Although phlebotomy has been reported to either increase [41,42,43] or have no significant effect on cerebral blood flow or cerebral blood FV [44], studies demonstrating increased CBF typically achieved a more robust reduction in haematocrit (approximately 4–5%) than observed in our study [42,43]. In contrast to phlebotomy, we found no published data examining the effect of rheopheresis on resting FV in the cerebral arteries.

### 4.3. Improved Visual Stimulation-Evoked Hemodynamic Responses After Rheopheresis but Not After Phlebotomy

To compare the FV data measured before and after interventions, absolute FV values were normalized to the baseline data in both MCA and PCA and expressed as relative FV in % of the appropriate baseline values.

While the relative FV changes evoked by visual stimulation were very similar before and after phlebotomy, the increase in the visually evoked relative FV was larger after rheopheresis compared to the pre-treatment values (Figure 2, Table 4). These findings indicate that rheopheresis was associated with a significant increase in the hemodynamic response evoked by neuronal activation, with no significant changes observed following phlebotomy.

Neuronal activation is typically coupled with increased cerebral blood flow, mediated through vasodilation of cerebral resistance vessels. This process can be augmented by the reduction in vascular resistance resulting from decreased blood viscosity following rheopheresis. However, in addition to the decrease in blood viscosity, other mechanisms may also contribute to the enhanced FV responses observed after rheopheresis. We agree with Klingel et al. [26], who suggested that removal of a spectrum of high-molecular-weight plasma proteins from human plasma not only decreases blood viscosity but also affects the complex interplay between plasma constituents, blood cells, vascular endothelium, and cellular and extracellular compartments of the surrounding tissue [45]. Furthermore, the elimination of LDL cholesterol that has been shown to inhibit NO synthase activity and inactivate endothelial-derived NO may also contribute to the improved vascular responses. Rubba et al. [46] and Weiss et al. [47] demonstrated that LDL removal led to an increase in peak blood flow to the calf and forearm during reactive hyperemia. Additional studies have shown that beyond the improvement in rheological properties after apheresis, removal of native and oxidized LDL in hypercholesterolemic patients enhanced the endothelium-dependent vasodilation [48] and improved both coronary vasodilatory capacity and cerebral CO_2_ reactivity [49,50].

Although a direct comparison between the rheopheresis and phlebotomy groups was not planned due to differences in age and vascular risk profiles, it is noteworthy that, prior to treatment, the flow velocity response to visual activation in the PCA was markedly lower in the rheopheresis group than in the phlebotomy group. This significant pre-treatment difference may reflect the less favorable risk factor profile in the rheopheresis group, which included a higher mean age and a greater prevalence of diabetes and hypercholesterolemia. Indeed, the neuronal activation-induced flow response has been shown to decline with age, and diabetes has been identified as the strongest risk factor for impaired neurovascular coupling in a large UK study [28]. It should be noted, however, that the two groups differed not only in the conventional vascular risk factors (age, diabetes, hypercholesterinaemia), but also in the hemorheological parameters: both plasma viscosity and whole-blood viscosity were higher in the rheopheresis group than in the phlebotomy group. Interestingly, despite the unfavorable risk factor profile in the rheopheresis group, the significant difference in the neuronal activation-induced FV response between the two groups disappeared after the treatment, which was due to the substantial improvement in neurovascular coupling after rheopheresis.

### 4.4. Results of VEP and BHI

Neurovascular coupling refers to the dynamic regulation of cerebral blood flow in response to neuronal activity, a process that involves the coordinated interaction of neurons, glial cells, and blood vessels, forming a functional unit called the neurovascular unit. To separately assess neuronal activation and vascular reactivity, the latency and amplitude of VEP and the flow velocity response to hypercapnia (breath-holding index) were measured. Both the amplitude and latency of the visually evoked potentials before and after treatment were similar in the phlebotomy and rheopheresis groups, indicating that neither intervention had a significant effect on occipital cortex activation.

Measurement of the breath-holding index demonstrated that the cerebral vasomotor reactivity in response to hypercapnia was within the normal range of 1.2 ± 0.4%/s [51] in both treatment groups, both before and after intervention. However, the BHI remained unchanged before and after phlebotomy, whereas it increased significantly following rheopheresis compared to the pre-treatment value. While we did not observe changes in cerebral vasoreactivity after phlebotomy, others have reported either decreased [52] or unchanged [53] cerebrovascular reactivity to hypercapnia following hemodilution. The improvement in cerebral vasoreactivity observed after rheopheresis is consistent with previous reports on CO_2_ reactivity following LDL apheresis [49]. In line with our findings, Pfefferkorn et al. [49] reported that a single session of heparin-mediated extracorporeal LDL precipitation, resulting in substantial removal of LDL cholesterol, Lp(a), and fibrinogen, led to an increase in cerebral CO_2_ reactivity from 22 ± 21% to 36 ± 18% in patients with coronary artery disease and hyperlipidemia. Similarly to our results, they did not observe changes in resting mean middle cerebral artery (MCA) flow velocity but measured a significantly greater CO_2_-induced flow velocity increase after treatment. This improvement was attributed to the normalization of endothelial dysfunction by LDL precipitation, as previously demonstrated also in peripheral [54] and coronary arteries [48]. A limitation of our study is that, while we focused on rheological parameters before and after intervention, we did not measure the pre- and post-treatment LDL cholesterol levels. Nevertheless, our findings suggest that rheopheresis significantly improves cerebral vasoreactivity, as indicated by the enhanced flow response to hypercapnia.

It should be highlighted that, unlike previous hemodilution studies conducted in unselected stroke populations, our study specifically included patients with elevated hematocrit or confirmed hyperviscosity. This distinction may be crucial, as hemorheological interventions are more likely to elicit measurable cerebrovascular effects in patients with pathological viscosity profiles.

### 4.5. Possible Causes of Improved Neurovascular Coupling After Rheopheresis

The improvement in cerebral vasoreactivity, in the absence of changes in VEP amplitudes after rheopheresis, suggests that the enhanced visually evoked FV increase, i.e., the improved neurovascular coupling, is attributable to an augmented vascular response rather than enhanced neuronal activation.

Although vasodilation is the final and essential step in both neurovascular coupling and cerebral vasomotor reactivity, an enhanced visually evoked flow response does not necessarily result from improved vasoreactivity, as the underlying regulatory mechanisms of these two processes are not completely identical. Previous human studies, however, have shown that the flow response to functional stimulation is attenuated under hypercapnic conditions compared to normocapnia [55,56,57], suggesting some overlap in the regulatory pathways involved in neurovascular coupling and cerebral vasoreactivity. The regulation of neuronal activation-induced flow response involves several signaling molecules, including nitric-oxide, arachidonic acid metabolites, adenosine, metabolic products (e.g., CO_2_, lactate), potassium ion, and neurotransmitters such as glutamate [58] while hypercapnia primarily regulates CBF through pH, nitric-oxide signaling and itself the pCO_2_ [59]. Since both neurovascular coupling and hypercapnia-induced vasodilations are at least partly mediated by nitric oxide [60,61,62,63,64,65], the enhancement of nitric oxide signaling after rheopheresis [46,47,48] may contribute to improved vasodilatory responses required for both neurovascular coupling and cerebral vasoreactivity.

### 4.6. Possible Role of Rheopheresis in Treatment of Patients with Cerebral Ischemia

According to current guidelines, hemodilution, including various methods of volume expansion with or without phlebotomy, is not recommended, as no randomized controlled trials have demonstrated its clear benefit, and multiple randomized controlled trials have failed to show significant positive effects of hemodilution in patients with acute ischemic stroke [66]. In line with this guideline, we did not observe any improvement following phlebotomy in resting cerebral blood FV or in the FV responses induced by hypercapnia or visual stimulation. However, while the effects of hemodilution have been extensively studied in unselected stroke populations, data on the impact of rheopheresis in ischemic stroke patients remain scarce.

Extracorporeal membrane differential filtration or extracorporeal rheopheresis has been reported to be both feasible and safe, with significant improvements in hemorheological parameters observed following treatment in patients with acute ischemic stroke [25,67]. However, no significant differences in neurological outcomes were found, and the prevalence of early reperfusion was similar to that in the control group not receiving rheopheresis [25]. In contrast, Walzl et al. [68] reported that two sessions of heparin-mediated extracorporeal LDL precipitation led to significant improvements in hemorheological parameters, which were associated with clinical improvement in 26 patients with acute embolic stroke and 22 patients with multi-infarct dementia compared to untreated controls. It should be noted, however, that due to the small sample sizes and observational nature of these studies, no definitive conclusions can be drawn regarding the clinical efficacy of rheopheresis in acute ischemic stroke.

It is also important to note that all previous phlebotomy and rheopheresis studies included unselected stroke populations, i.e., patients without erythrocytosis, polycythemia, or elevated blood viscosity. Therefore, no conclusions can be drawn regarding the effectiveness of hemorheological interventions in stroke patients with pathological rheological profiles. As our study demonstrated improved vasoreactivity and enhanced neuronal activation-evoked flow responses following rheopheresis in patients with elevated blood viscosity, it would be logical and clinically relevant to investigate the impact of rheopheresis on outcomes in ischemic stroke patients with abnormal hemorheological parameters.

### 4.7. Limitations

This study has several limitations. First, the phlebotomy and rheopheresis groups differed substantially in baseline characteristics, including age and the prevalence of vascular risk factors such as diabetes mellitus and hypercholesterolemia. Moreover, group allocation was based on clinical indication, resulting in two distinct patient populations with different underlying pathophysiological conditions (erythrocytosis vs. hyperviscosity-related disorders). Therefore, direct comparisons between the two interventions should be interpreted with caution, and the findings should primarily be regarded as reflecting within-group effects, as the study was not designed or powered to determine the superiority of one treatment over the other. Second, the sample size in both groups was relatively small, limiting the statistical power and generalizability of the findings. Importantly, despite this limitation, a statistically significant within-group effect was observed in the rheopheresis group. Nevertheless, given the small sample size and the absence of long-term follow-up data, our results should be interpreted carefully. Larger, longitudinal studies are needed to confirm the durability and clinical relevance of the hemodynamic improvements observed after rheopheresis. Third, cerebral hemodynamics were assessed using TCD, which measures absolute flow velocities that are not directly proportional to cerebral blood flow (CBF) across individuals. To account for this limitation, we focused on relative changes in flow velocity induced by hypercapnia or visual cortex activation. These relative changes are known to correlate well with relative changes in CBF, under the assumption that the diameter of the insonated artery remains constant during stimulation [69]. Fourth, although efforts were made to ensure consistency in probe positioning (same examiner, insonation depth, and acoustic window), repositioning between sessions may have introduced measurement variability. Future studies using neuronavigation-assisted TCD or multimodal imaging could further minimize this potential source of error. Fifth, we assessed the visual stimulation-induced FV response in the PCA and the hypercapnia-induced hemodynamic response in the MCA. The MCA was selected for evaluating vasomotor reactivity because established reference values for the breath-holding index (BHI) are available for this artery. Due to known regional differences in cerebrovascular reactivity [70], a direct comparison between the hypercapnia-induced FV changes in the anterior circulation and the visual stimulation-evoked FV responses in the posterior circulation is not appropriate. Finally, cerebral vasoreactivity was assessed using the BHI, a method that has certain limitations, including the involvement of the Valsalva maneuver during breath holding. To minimize the influence of increased intrathoracic pressure during breath-holding, participants were instructed to perform the test without using Valsalva maneuver. Additionally, to reduce variability in breath-hold duration, all participants were asked to hold their breath for a fixed duration of 30 s.

## 5. Conclusions

Our data indicate that rheopheresis reduces both whole-blood and plasma viscosity in patients with hyperviscosity and leads to a significant improvement in cerebral vasoreactivity and neurovascular coupling without affecting visual evoked potential (VEP) parameters. The observed enhancement in cerebral blood flow response to visual stimulation, in the absence of changes in VEPs, suggests that the improvement in neurovascular coupling is likely attributable to improved vascular responsiveness rather than increased neuronal activation in the occipital cortex. In contrast, similar improvements in hypercapnia- or visual stimulation-induced flow responses were not observed following phlebotomy in patients with erythrocytosis. The observed improvement in cerebral vasoreactivity and neurovascular coupling suggests that rheopheresis may represent a targeted therapeutic strategy for patients with hyperviscosity-associated cerebrovascular dysfunction. Although the sample size was relatively small and long-term follow-up data are lacking, the robust effects of rheopheresis on cerebral hemodynamics highlight the need for further controlled trials to determine whether these hemodynamic improvements translate into meaningful neurological or cognitive benefits and to assess the clinical relevance of this intervention in hyperviscosity-related cerebrovascular disorders.

## Figures and Tables

**Table 1:** Characteristics of the patients at enrollment.

	Rheopheresis (*n* = 9)	Phlebotomy (*n* = 11)
Age, years	64.2 ± 5.5	53.7 ± 12.8 *
Sex (male), *n* (%)	8 (88.89)	11 (100.00)
Hypertension, *n* (%)	8 (88.89)	6 (54.55)
Diabetes mellitus, *n* (%)	8 (88.89)	2 (18.18) *
Smokers, *n* (%)	1 (11.11)	2 (18.18)
Hypercholesterolemia, *n* (%)	7 (77.78)	2 (18.18) *
Atrial fibrillation, *n* (%)	0 (0)	0 (0)
Systolic blood pressure (Hgmm) before treatment	134 ± 7	131 ± 7
Systolic blood pressure (Hgmm) after treatment	133 ± 6	130 ± 5
Diastolic blood pressure (Hgmm) before treatment	84 ± 4	81 ± 5
Diastolic blood pressure (Hgmm) after treatment	83 ± 4	80 ± 4
Pulse rate (1/min) before treatment	74 ± 4	73 ± 5
Pulse rate (1/min) after treatment	75 ± 5	72 ± 5

* *p* < 0.05 (ANOVA).

**Table 2:** Laboratory parameters before and after intervention.

	Rheopheresis			Phlebotomy		
	Before Treatment	After Treatment	*p* Value	Before Treatment	After Treatment	*p* Value
WBC, 10^9^/L	6.37 ± 1.28	6.56 ± 1.08	0.62	7.37 ± 2.25	7.22 ± 2.15	0.69
RBC, 10^12^/L	5.16 ± 0.69	4.88 ± 0.66	0.09	6.20 ± 0.35	5.75 ± 0.34	0.01
Hgb, g/L	152.83 ± 16.93	145.94 ± 21.28	0.10	176.00 ± 7.93	166.25 ± 10.31	0.01
Hct, %	43.75 ± 5.38	42.38 ± 6.43	0.29	51.51 ± 1.68	49.60 ± 2.28	0.01
MCV, fL	85.24 ± 4.37	86.47 ± 3.76	0.23	85.20 ± 4.00	86.03 ± 3.65	0.12
MCH, pg	29.87 ± 1.56	29.93 ± 1.58	0.80	29.46 ± 2.40	29.52 ± 2.37	0.91
MCHC, g/L	351.44 ± 10.45	346.17 ± 8.12	0.13	345.59 ± 18.86	342.77 ± 16.42	0.62
PLT, 10^9^/L	233.72 ± 77.41	204.89 ± 51.10	0.08	219.59 ± 78.53	218.64 ± 77.80	0.89
WBV, mPas	5.68 ± 0.80	4.48 ± 0.90	0.002	5.60 ± 0.62	5.22 ± 0.89	0.16
PV, mPas	1.53 ± 0.13	1.31 ± 0.15	0.002	1.35 ± 0.19	1.33 ± 0.14	0.79
WBV at 40% hematocrit, mPas	5.34 ± 1.09	4.32 ± 0.29	0.02	4.36 ± 0.49	4.22 ± 0.58	0.28
Hematocrit/WBV ratio	7.83 ± 1.38	9.57 ± 0.98	0.003	8.79 ± 0.98	9.37 ± 1.61	0.23
Red blood cell deformability						
EI 3Pa	0.32 ± 0.01	0.31 ± 0.02	0.11	0.30 ± 0.04	0.29 ± 0.04	0.59
EI_max_ (SS)	0.64 ± 0.02	0.61 ± 0.04	0.10	0.62 ± 0.04	0.63 ± 0.04	0.48
SS_1/2,_ Pa	3.26 ± 0.46	3.16 ± 0.64	0.72	3.36 ± 0.98	3.76 ± 0.98	0.26
EI_max_/SS_1/2,_ 1/Pa	0.20 ± 0.02	0.20 ± 0.03	0.99	0.20 ± 0.05	0.18 ± 0.05	0.19
EI min	0.12 ± 0.01	0.12 ± 0.01	0.44	0.14 ± 0.03	0.14 ± 0.03	0.45
EI_max_ (osm)	0.55 ± 0.01	0.55 ± 0.01	0.42	0.55 ± 0.01	0.55 ± 0.01	0.39
EI hyper	0.28 ± 0.01	0.28 ± 0.003	0.74	0.27 ± 0.01	0.28 ± 0.01	0.36
O min, mOsm/kg	140.00 ± 5.81	141.89 ± 3.37	0.38	135.36 ± 6.64	134.10 ± 8.10	0.49
O (EI) max, mOsm/kg	284.67 ± 11.44	290.11 ± 10.12	0.02	270.82 ± 14.53	276.09 ± 16.23	0.22
Ohyper, mOsm/kg	420.56 ± 9.86	418.67 ± 4.77	0.40	410.64 ± 19.32	415.55 ± 22.11	0.87
Area, au	144.82 ± 7.18	143.61 ± 5.70	0.72	139.70 ± 11.38	138.13 ± 16.20	0.73
Red blood cell aggregation						
M 5 s	3.93 ± 0.80	2.39 ± 1.45	0.04	2.71 ± 0.96	3.16 ± 0.72	0.16
M1 5 s	3.54 ± 1.32	2.43 ± 1.32	0.10	2.88 ± 1.35	2.45 ± 1.15	0.27
M 10 s	9.10 ± 5.23	7.19 ± 5.40	0.50	7.97 ± 3.20	7.62 ± 3.77	0.65
M1 10 s	8.96 ± 5.19	5.65 ± 4.12	0.25	7.37 ± 3.47	7.23 ± 2.88	0.84
AI, %	84.76 ± 11.03	60.59 ± 31.33	0.041	77.10 ± 11.59	79.19 ± 9.88	0.55
Amp, au	14.81 ± 2.62	9.19 ± 5.24	0.004	8.64 ± 3.65	11.37 ± 2.40	0.42
t_1/2_, s	0.75 ± 0.68	1.62 ± 2.67	0.40	1.59 ± 0.94	1.35 ± 0.77	0.41
isc_1/2_, au	12.64 ± 4.52	11.71 ± 4.34	0.44	11.23 ± 5.78	9.91 ± 3.00	0.64

WBC—White blood cell count; RBC—Red blood cell count; Hgb—hemoglobin; Hct—hematocrit; MCV—Mean corpuscular volume; MCH—Mean corpuscular hemoglobin; MCHC—Mean corpuscular hemoglobin concentration; PLT—Platelet count; WBV—Whole-blood viscosity; PV—Plasma viscosity; EI 3Pa—elongation index at a shear stress of 3 Pascal; EI_max_ (SS)—maximal elongation index under increasing shear stress; SS1/2—shear stress at the half of the maximal elongation index; EImin—minimal elongation index at low osmolality; EI_max_ (osm)—maximal elongation index under increasing osmolality; EI hyper—elongation index in the hyper-osmotic environment; Omin—osmolality at which hemolysis begins; O(EI)max—osmolality where maximal deformability occurs; Ohyper—osmolality at which elongation index in the hyperosmotic region is measured; Area—the area under the elongation index–osmolality curve; M 5 s and M 10 s—aggregation of red blood cells at a zero shear rate value in the 5th and 10th seconds—M1 5 s and M1 10 s—aggregation of red blood cells at a low shear rate value in the 5th and 10th seconds; AI—aggregation index; Amp—amplitude; t_1/2_—half time of aggregation_;_ isc_1/2_—slope index at half amplitude.

**Table 3:** Absolute baseline (resting) flow velocity parameters in the posterior and middle cerebral arteries before and after interventions.

	Rheopheresis			Phlebotomy		
Baseline Absolute Flow Velocity Values	Before Treatment	After Treatment	*p* Value	Before Treatment	After Treatment	*p* Value
MCA TAMV (cm/s)	46.16 ± 8.55	45.62 ± 8.36	0.81	48.80 ± 11.32	47.78 ± 7.08	0.65
PCA PSV (cm/s)	44.75 ± 6.26	44.72 ± 4.46	0.985	39.18 ± 8.99	38.66 ± 9.90	0.801
PCA TAMV (cm/s)	24.87 ± 5.54	24.49 ± 3.96	0.794	24.34 ± 6.53	24.28 ± 7.49	0.965

MCA: middle cerebral artery; PCA: posterior cerebral artery; PSV: peak-systolic flow velocity; TAMV: time-averaged mean flow velocity. Data before and after treatment were compared using a paired *t*-test. Data are mean ± standard deviation.

**Table 4:** Maximum relative flow velocities measured in the posterior cerebral artery during visual stimulation, before and after treatment.

	Rheopheresis			Phlebotomy		
	Before Treatment	After Treatment	*p* Value	Before Treatment	After Treatment	*p* Value
Maximum relative PSV (%)	113.90 ± 3.98	119.54 ± 6.39	0.017	119.70 ± 5.41	120.97 ± 8.08	0.338
Maximum relative TAMV (%)	117.64 ± 6.91	125.09 ± 7.47	0.001	127.13 ± 9.87	128.43 ± 10.81	0.278

PSV: peak systolic flow velocity; TAMV: time-averaged mean flow velocity. Data before and after treatment were compared with paired *t*-test. Data are mean ± standard deviation.

## Data Availability

The raw data supporting the conclusions of this article will be made available by the authors on request.

## Abbreviations

The following abbreviations are used in this manuscript:

AI	Aggregation index.
Amp	Amplitude.
Area	The area under the elongation index–osmolality curve.
BHI	Breath-holding index.
CBF	Cerebral blood flow.
EI 3Pa	Elongation index at a shear stress of 3 Pascal.
EI hyper	Elongation index in the hyper-osmotic environment.
EImax osm	Maximal elongation index under increasing osmolality.
EImax SS	Maximal elongation index under increasing shear stress.
EImin	Minimal elongation index at low osmolality.
FV	Flow velocity.
Hct	Hematocrit.
Hgb	Hemoglobin.
isc_1/2_	Slope index at half amplitude.
ISCEV	International Society for Clinical Electrophysiology of Vision.
MCA	Middle cerebral artery.
MCH	Mean corpuscular hemoglobin.
MCHC	Mean corpuscular hemoglobin concentration.
MCV	Mean corpuscular volume.
O(EI)max	Osmolality where maximal deformability occurs.
Ohyper	Osmolality at which elongation index in the hyperosmotic region is measured.
Omin	Osmolality at which hemolysis begins.
PCA	Posterior cerebral artery.
PI	Pulsatility index.
PLT	Platelet count.
PSV	Peak systolic flow velocity.
PV	Plasma viscosity.
RBC	Red blood cell count.
SS1/2	Shear stress at the half of the maximal elongation index.
t_1/2_	Half time of aggregation.
TAMV	Time-averaged mean.
TCD	Transcranial Doppler.
VEP	Visual evoked potential.
WBC	White blood cell count.
WBV	Whole-blood viscosity.
